# Replication of Radial Pulses Using Magneto-Rheological Fluids

**DOI:** 10.3390/mi15081010

**Published:** 2024-08-06

**Authors:** Miranda Eaton, Jeong-Hoi Koo, Tae-Heon Yang, Young-Min Kim

**Affiliations:** 1 Department of Mechanical and Manufacturing Engineering, Miami University, Oxford, OH 45056, USA; eatonml4@miamioh.edu (M.E.); koo@miamioh.edu (J.-H.K.); 2 Department of Mechanical Engineering, Konkuk University, Seoul 05029, Republic of Korea; 3 Digital Health Research Division, Korea Institute of Oriental Medicine, 1672 Yuseong-daero, Yuseong-gu, Daejeon 34054, Republic of Korea

**Keywords:** magneto-rheological fluids, radial pulses, pulse waveforms, pulse simulation

## Abstract

The radial pulse is a critical health marker with expanding applications in wearable technology. To improve these applications, developing a pulse generator that consistently produces realistic pulses is crucial for validation and training. The goal of this study was to design and test a cost-effective pulse simulator that can accurately replicate a wide range of age-dependent radial pulses with simplicity and precision. To this end, this study incorporated a magneto-rheological (MR) fluid device into a cam-based pulse simulator. The MR device, as a key component, enables pulse shaping without the need for additional cams, substantially reducing the cost and complexity of control compared with existing pulse simulators. To evaluate the performance of the MR pulse simulator, the root-mean-square (RMS) error criterion (less than 5%) was used to compare the experimentally obtained pulse waveform with the in vivo pulse waveform for specific age groups. After demonstrating that the MR simulator could produce three representative in vivo pulses, a parametric study was conducted to show the feasibility of the slope-based pulse-shaping method for the MR pulse simulator to continuously generate a range of age-related pulses.

## 1. Introduction

The radial pulse has long served as a vital aspect of healthcare, offering insights into an individual’s health status [1,2,3,4]. In Western medicine, the augmentation index and augmentation pressure derived from radial pulse pressure waveforms have emerged as reliable markers for assessing arterial compliance, cardiovascular disease risk, and overall health [1,2,5,6,7]. Similarly, Oriental Medicine (OM) and Tactical Combat Casualty Care (TCCC) have relied on palpating radial pulse pressures with three fingers as a diagnostic tool [8,9,10]. The three-finger technique is a well-known and long-standing method of diagnosis in OM, where the physician places three fingers over the patient’s radial artery to assess the patient’s health condition. A physician applies differing levels of pressure to ascertain the medical state of the patient [9]. While pulse diagnosis is a standard practice in OM, it heavily relies on the expertise and subjectivity of physicians. The readings of a pulse can, indeed, be highly subjective, varying from person to person. Therefore, there is a significant opportunity to enhance the teaching and training of pulse diagnosis in OM by standardizing common pulses. By establishing standardized guidelines, the consistency and accuracy of pulse diagnosis can be greatly improved. Furthermore, the radial pulse serves as an indicator for TCCC on the battlefield, as mentioned in the *TCCC Handbook* [11]. The *TCCC Handbook* provides instructions for medical care in combat situations, where the strength of the radial pulse is used to assess the condition of a wounded soldier, particularly to determine if they are in a state of shock. If an “abnormal radial pulse” is observed, the wounded soldier is considered to be in shock, and the appropriate amount of fluid is administered [11]. However, the term “abnormal” can be vague and inconsistent among soldiers. Thus, the implementation of a standardized approach to measuring pulses could help in training soldiers to accurately assess wounded individuals on the battlefield.

The radial pulse can play a vital role in the wearable healthcare industry. The rise of wearable technology has brought about a revolution in healthcare by enabling continuous real-time monitoring of vital signs, empowering users with a better understanding of their well-being. Wrist-worn systems, in particular, strive to offer non-invasive real-time blood pressure (BP) monitoring through sensor readings from radial pulses. This technology facilitates convenient and accessible blood pressure checks, potentially resulting in enhanced health outcomes.

Advancing the technology and medical applications related to the radial pulse requires the development of pulse simulators that can generate a wide array of human radial pulses. These simulators are essential for calibrating wearable sensors and training medical professionals in pulse palpation techniques. While the subjective nature of the three-finger technique used in OM underscores the need for pulse standardization, wearable healthcare devices require certified and calibrated embedded sensors to ensure the quality and reliability of collected data [5]. Clinical trials are a valuable means to validate sensor accuracy and provide training for medical personnel. However, it is important to acknowledge that human subject testing can be both expensive and time-consuming. Furthermore, the variability in blood pressure in patients throughout the day adds another layer of complexity [12]. Therefore, while clinical trials remain crucial, they can be resource-intensive due to the involvement of human subjects, rendering pulse simulators a cost-effective and dependable alternative for validating and calibrating wearable technology.

Currently, a few pulse generators are available on the market. Commercially available pulsatile blood pumps are designed to simulate animal pulses, while other simulators cater to medical professional training [13,14]. These pulse generators have limited capacity to generate user-defined pulse waveforms and tend to be bulky. Ongoing research is focused on developing more advanced and versatile simulators. One study presented a model that incorporates all four chambers of the heart to allow for tunable stiffness [15]. While the article found the set-up can closely match the waveforms of various heart conditions, it did not investigate replicating a range of age-related pulses [15].

To enhance the versatility and capabilities of pulse generation, another study employed smart or controllable fluids in a pulse simulation system [16]. This system utilized a peristaltic pump to circulate a magneto-rheological (MR) fluid whose properties could be controlled by an external magnetic field. As a class of smart materials, MR fluids have been utilized in various engineering applications, including automotive suspension systems and haptic devices [17,18,19,20,21,22,23,24,25,26,27]. An MR fluid consists of micron-sized iron particles suspended in a carrier fluid [28]. These particles respond to an external magnetic field by resisting flow, with a resistance proportional to the field’s strength. Consequently, MR fluids are highly controllable, exhibit rapid response times, and require low power for operation. However, MR fluid-based systems typically need an electromagnet to generate the magnetic field, which poses challenges for miniaturizing MR devices. In a peristaltic pump system, a magnetic valve was used to control the fluid motion to create pulse pressure waveforms. By manipulating the peak amplitude ratio and separation or time delay between two peaks, various “two-peak” pulse waveform patterns were created, demonstrating the controllability of the augmentation index. Another investigation involved the development of a small pulsatile simulator using a cam-follower system [29]. A specially designed disk cam, generated from in vivo radial pulse data, replicated pulse waveforms accurately and consistently. However, this cam-based pulse simulator required the fabrication of a new cam disk for each desired pulse waveform, rendering it unable to continuously generate a range of radial pulses.

In a feasibility study conducted by Eaton et al., an MR pulse-shaping method was employed for a cam-based pulse system in order to generate a variety of pulse waveforms [30]. The MR fluid was utilized to shape a base pulse into a desired waveform, successfully replicating age-specific pulse waveforms using multiple cam disks. While the study demonstrated the feasibility of the MR fluid system in shaping age-related pulses, further enhancements are required to achieve a wide range of target pulse waveforms without having to use multiple cams.

The present study aimed to expand upon the aforementioned feasibility study by replicating a range of age-related pulses using a single cam. The primary goal of the “MR fluid based blood pulse-shaping” technique is to eliminate the need for multiple cam disks for different age groups, enabling the continuous and repeatable generation of pulses in a cost-effective manner. A single baseline pulse created by a cam pulse generator is shaped into a variety of age-related radial pulse waveforms using an MR fluid as the means to shape the baseline pulse. Furthermore, a parametric study will be presented, which further investigates the relationship between the input duty values and the resulting waveform. Two cams were utilized in this paper, an MR shaping cam and a half-cam. The MR shaping cam was used to shape the pulses into the desired in vivo waveforms, while the half-cam was used during the parametric study.

The outline of this paper begins with the experimental set-up and the approach to shaping cams via an MR fluid. It then transitions to the discussion and results of shaping a base pulse into three distinct ages using a single cam. Finally, the parametric study is discussed, explaining in detail the relationship between the input duty values and the output slope of the waveform.

## 2. MR Pulse-Shaping Design and Technique

### 2.1. Experimental Set-Up

**Figure 1:**
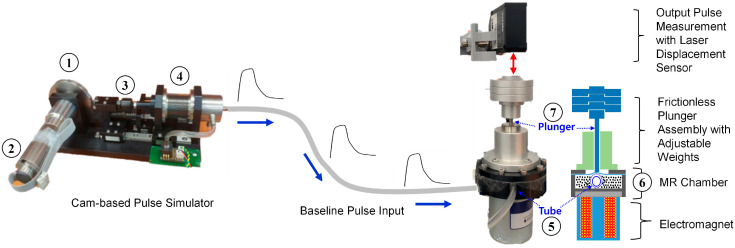
Illustration of experimental set-up, including cam pulse generator, electromagnet, frictionless plunger assembly, and displacement sensor.

The experimental set-up is shown in Figure 1. It primarily consisted of the cam-based pulse simulator and a pulse-shaping module based on a magneto-rheological (MR) fluid. The key components of the design included a cam pulse generator, an electromagnet, an MR fluid chamber, and a plunger assembly. A thorough discussion of the design and construction of the cam-based simulator can be found in [31]; however, for the purpose of this work, a concise explanation is presented here. As shown in Figure 1, the cam (①) was designed by converting an in vivo radial pulse waveform into polar coordinates so that its profile represented an actual human radial pulse waveform. One full revolution of the cam produced one period of a radial pulse waveform. The cam was precisely fabricated from stainless steel 304 using wire-cutting technology. As shown in Figure 1, the cam was connected to a DC motor (②; Maxon Motor, DCX 26L), which regulated the beats per minute (bpm) of the pulse by controlling the rotational speed of the cam. To track the cam’s rotational position, a Hall effect sensor (Honeywell, SS541AT) was placed between the DC motor and the cam. This sensor read the magnetic field from a small permanent magnet mounted on the cam as it rotated, providing critical timing information for the electromagnet control in the MR chamber. As the cam pushed against the cam-follower assembly, which was installed on a linear guide (③), it compressed or extended the ultra-soft bellows module (④) to generate a base pulse. This base pulse was transmitted to the MR fluid chamber (④) via an elastic tube (⑤) representing a blood vessel. The MR fluid chamber (⑥) sat on top of the electromagnet, with a Gauss meter probe placed between the two to measure the magnetic field generated by the electromagnet. The plunger (⑦) assembly, shown in Figure 1, consisted of a frictionless piston and cylinder, with weights placed on top of the piston to allow for tunable “hold-down pressure”. The frictionless plunger was placed directly above the MR chamber to measure the displacement. A detailed explanation of the plunger design is provided in reference [16]. As the base pulse was sent to the MR chamber, it caused the tube to expand, moving the plunger along with the base pulse. The plunger placed on the soft tube moved up and down depending on the degree to which the tube was expanded by the pulse input. A laser sensor placed above the plunger head measured this displacement. This displacement represented the pulse waveform, which was then shaped into a variety of pulses.

**Figure 2:**
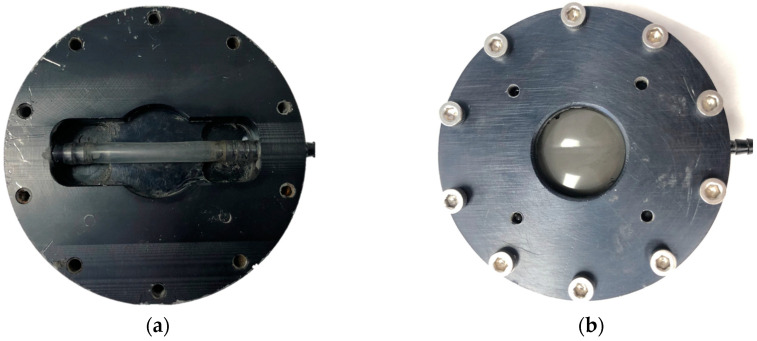
(**a**) Empty MR fluid chamber with exposed silicone tubing; (**b**) fully assembled chamber filled with MR fluid and covered with film and lid.

A detailed description of the MR chamber is provided below and illustrated in Figure 2. Figure 2a shows the empty MR chamber, revealing the silicone tube inside that represents the radial artery. The base pulse was sent from the cam generator to this silicone tube inside the chamber. The chamber was then filled with the MR fluid, which completely surrounded the silicone tube. A flexible membrane was placed over the chamber to prevent any excess fluid from spilling out. The top of the chamber was placed over the membrane and secured with bolts. Figure 2b depicts the fully assembled MR chamber, which was 64 mm in diameter and 28 mm in height with the cover. The MR fluid shaped the base pulse via the electromagnet sitting beneath the chamber. When the MR fluid surrounding the soft tube was activated by an electromagnetic field, it restricted the expansion of the soft tube caused by the base pulse input. This restriction limited the external displacement and facilitated precise pulse shaping. Consequently, the MR fluid effectively controlled the expansion of the tubing within the chamber, allowing the base pulse to be shaped into a wide range of pulse profiles.

### 2.2. Working Principle

The following section discusses the procedure behind shaping the pulse using an MR fluid. To perform MR shaping, the magnetic field generated by an electromagnet controls the state of the MR fluid. Therefore, the precise generation of this field is crucial. To achieve this, our study employed pulse width modulation (PWM) to adjust the output magnetic field of the electromagnet. PWM is a technique used to control analog actuators with a digital signal. It regulates the average value of a waveform over a switching period by adjusting the pulse width or duty cycle.

In this study, we controlled the output electromagnetic field strength of the analog electromagnet by varying the pulse width at a frequency of 500 Hz. The duty cycle of the PWM signal, expressed as a percentage, determined the proportion of time the signal was on, thereby controlling the electromagnet’s output.

**Figure 3:**
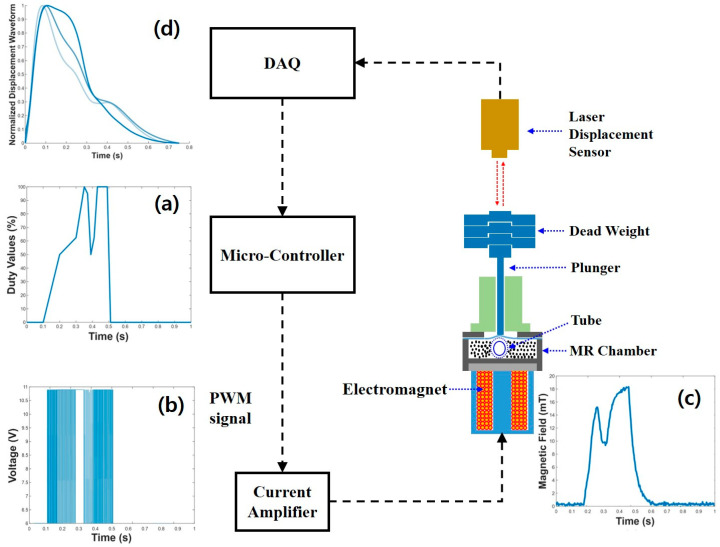
(**a**) Input duty values programmed in the micro-controller; (**b**) pulse width modulation (PWM) signal generated by the micro-controller; (**c**) resulting magnetic field from input PWM signal; and (**d**) age-dependent pulse generation measured by the laser displacement sensor.

The steps to control the MR fluid are shown in Figure 3. Figure 3a illustrates the duty cycle values input to the Arduino unit. These values ranged from 0 to 100% and could be set to operate for a user-specified duration. Given that one cycle of an average pulse is approximately 750 ms, the duty cycle profile was adjusted within this 750 ms timeframe. As previously explained, the Hall effect sensor mounted on the cam system detected the rotational motion of the cam dis. The duty cycle profile was controlled to synchronize with the cam’s rotation, ensuring that the changes occurred repeatedly in sync with the cam’s rotation. Figure 3b shows the sample PWM signals, which were created from the duty values. Essentially, the duty values accounted for the percentage of time the signal was on, which could range from 0 to 100%. The adjustable delay time was useful to start the duty at the desired time. Once the Arduino code was executed and the driver board for the electromagnet produced the PWM signals, those signals were then sent to the electromagnet to generate a magnetic field, as shown in Figure 3c. The strength of the magnetic field was determined by the magnitude of the duty values, which controlled the PWM inputs. Once a magnetic field was present, the MR fluid in the chamber was activated and changed the stiffness of the environment surrounding the silicone tube in the chamber. The MR fluid then changed the expansion of the silicone tube, which changed the amount the plunger moved vertically. This change in vertical displacement was measured using the laser sensor, which represented the generated pulse waveform, as shown in Figure 3d. The displacement (i.e., pulse waveform) was then matched to in vivo pulses to determine the accuracy of the system. For the first experimental study, the goal was to match the base pulse to the three in vivo pulses in Figure 3d, corresponding to the ages of 20, 50, and 80 years old. In this study, the radial artery pulse wave data of humans according to age, measured using the Robot Blood Pressure Measurement System (RTS) of the Korea Institute of Oriental Medicine, were adopted for a comparison study [32]. Therefore, the objective of this study was to match the experimentally generated pulses, as measured by the displacement of the plunger head, to various age-related in vivo pulses.

## 3. Experimental Evaluation of Pulse Shaping

One of the main objectives of this study was to utilize a single cam in the pulse simulator to generate a diverse set of radial pulse waveforms. Unlike using multiple disks, the proposed cam pulse generator can effectively reproduce a range of radial pulses with a single cam disk. This approach enhances system versatility and reduces costs by eliminating the need for multiple disks and the associated design and fabrication processes. This section focuses on reproducing age-dependent pulse waveforms using the MR pulse simulator with a single baseline cam. The sample in vivo pulse waveforms are compared with those generated by the MR simulator with a single cam, and an error analysis to assess its performance is performed.

### 3.1. Single-Cam Pulse Shaping

**Figure 4:**
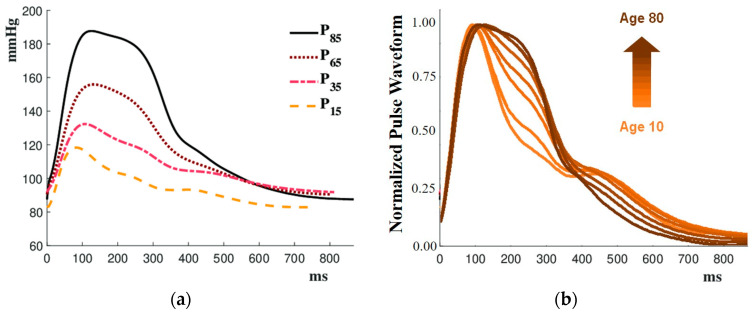
(**a**) Example of range of age-related in vivo radial pulse waveforms; (**b**) normalized pulse waveforms for ages 10–80.

Figure 4a, reproduced from [32], shows the range of age-dependent in vivo radial pulse waveforms. Younger pulses exhibit a sharp initial peak during systole with multiple additional peaks during diastole. As age increases, the initial peak widens, and the later portion of the pulse smooths out. To facilitate comparison, the pulses were normalized, as shown in Figure 4b. This figure illustrates that with age, the pulse magnitude increases, and the initial peak becomes wider. The steep incline of the first peak remains nearly identical across all ages. The trend with aging shown in the normalized graph in Figure 4b is also explained in the same way in the study of human arterial pulse waveforms according to age by Kelly et al. [33]. They reported that with age, the velocity of the reflected wave increases, the peaks of the radial arterial pulse waveform gradually converge, and the amplitude increases. Generating pulses with these age-related variations and producing multiple cam disks is time-consuming and costly. Therefore, this study intended to reproduce these pulses using the MR pulse simulator that employs a single cam.

**Figure 5:**
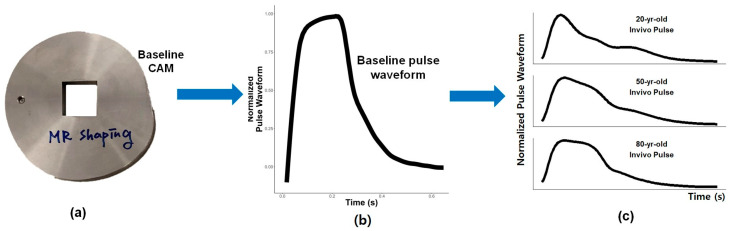
(**a**) MR pulse-shaping cam; (**b**) pulse waveforms generated by baseline cam for (**c**) 20, 50, and 80-year-old normalized waveforms into which the base pulse waveform is shaped.

The present study focused on three specific ages to illustrate the lifespan of a human. The three chosen pulse waveforms were 20, 50, and 80 years old. These waveforms exhibit distinct variations associated with age-related pulses, serving as representative examples across the human lifespan. Figure 5a shows the single cam used in this study, and the base pulse generated from the cam is displayed in Figure 5b. The cam produced a waveform representing an elderly pulse pattern, which was modified to simulate the pulse waveforms of younger individuals. The cam’s design resembles the pulse waveform of an 80-year-old because it is more straightforward to compress a pulse than to expand it. In other words, it is easier to create the diverse peaks of a 20-year-old pulse from an 80-year-old pulse than vice versa. Consequently, the experimental data collected will be compared with the normalized in vivo pulse waveforms shown in Figure 5c to evaluate the effectiveness of using an MR fluid to shape a base pulse.

### 3.2. Results of Single-Cam Pulse Shaping

**Figure 6:**
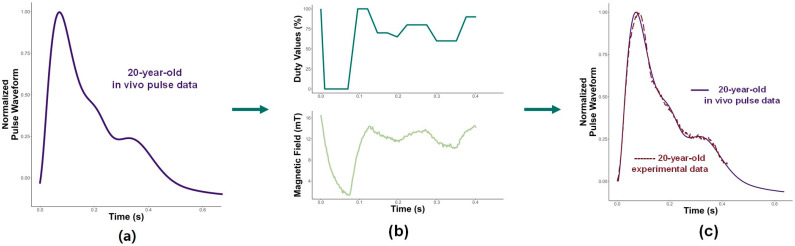
(**a**) Target 20-year-old in vivo pulse to be shaped; (**b**) duty values and magnetic field used to shape the base pulse into a 20-year-old pulse; and (**c**) resulting displacement and experimental displacement compared with 20-year-old in vivo pulse.

Figure 6a shows the first target in the vivo pulse waveform for a 20-year-old. Figure 6b displays the corresponding duty values (top) and the magnetic field (bottom) used to shape the base pulse. The duty values closely match the shape of the magnetic field, reinforcing the previously determined 1:1 relationship between the duty values and the magnetic field. However, an inverse relationship between the magnetic field and displacement can be observed. Moreover, Figure 6b shows that the duty values (and, consequently, the magnetic field) exhibit three distinct peaks, similar to the three peaks seen in the 20-year-old in vivo pulse waveform.

Figure 6c compares the shaped base pulse (dotted line) with the normalized in vivo pulse for a 20-year-old (solid line). While a typical pulse waveform lasts 0.75 s, the experimental results show shaping up to 0.4 s, due to the current method of pulse shaping. Nonetheless, any differences beyond 0.4 s would be negligible and imperceptible to humans. The most important section of the pulse is the first 0.4 s, including the initial peak. The initial ascent and sharp descent of the first peak in the experimental data closely match those of the in vivo pulse, typical of a young pulse. Additionally, the peaks in the diastolic section are nearly identical to the in vivo waveform. Given the close alignment between the experimental results and the in vivo waveform, the method effectively shaped the pulse to replicate that of a 20-year-old.

**Figure 7:**
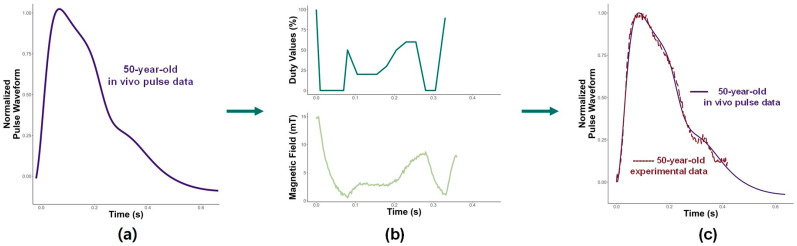
(**a**) Target 50-year-old in vivo pulse to be shaped; (**b**) duty values and magnetic field used to shape the base pulse into a 50-year-old pulse; and (**c**) resulting displacement and experimental displacement compared with 50-year-old in vivo pulse.

Since the method was effective at shaping a younger pulse, a middle-aged pulse was tested next. Figure 7 shows the duty values, magnetic field, and displacement used to shape the base pulse into a 50-year-old in vivo pulse. The duty values are not as large for the 50-year-old as they are for the 20-year-old pulse-shaping. This is attributed to the higher magnitude of displacement in older pulse waveforms. As one ages, the pulse waveform has higher pressure. Because of this increase in the magnitude of the waveform, less magnetic strength is required to constrict the expansion of the tube within the MR chamber. As a result, lower duty values are needed. Additionally, there are about two peaks observed in a 50-year-old pulse, which is then shown in the input duty values. The less-steep descent of the 50-year-old initial peak is displayed in the duty values, which return to 50% at 0.1 s instead of 100%, as was observed in the 20-year-old pulse. The plateau of the duty values between 0.1 s and 0.2 s resulted in a steady descent in the displacement.

Comparing the experimental displacement with the 50-year-old in vivo waveform, it can be noticed how they are very similar in Figure 7c. As with the 20-year-old, the initial peak is matched well. While the sharp ascent is similar to the younger pulse, the descent is more gradual and occurs over a longer period of time. The system can mimic the longer initial peak and the steady decline of the waveform. The smaller peaks during diastole are matched well and show the MR shaping technique is also capable of shaping a middle-aged adult pulse.

**Figure 8:**
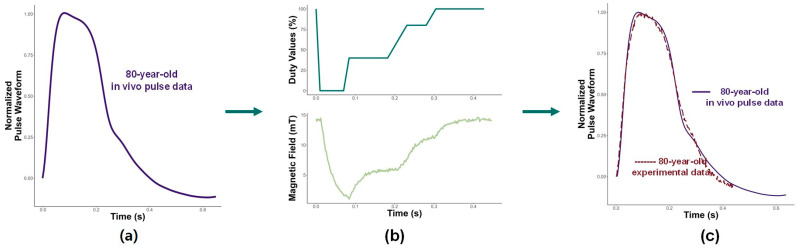
(**a**) Target 80-year-old in vivo pulse to be shaped; (**b**) duty values and magnetic field used to shape the base pulse into an 80-year-old pulse; and (**c**) resulting displacement and experimental displacement compared with 80-year-old in vivo pulse.

It was established in the previous two tests that the single-cam method can shape young and middle-aged pulses. The final test determined the ability of the system to replicate an elderly pulse. Therefore, the last age the study shaped was an 80-year-old pulse. Figure 8 shows the duty values and magnetic field required to regenerate the pulse. The main difference here is the elimination of any peaks in the duty values. As mentioned previously, elderly pulses do not exhibit any additional peaks during diastole. Because of this, the duty values gradually increase, which leads to the magnetic field slowly growing. The inverse of a slowly increasing magnetic field is shown in the displacement with a gradual decline.

The results of comparing the experimental displacement with an 80-year-old in vivo pulse are shown in Figure 8. An 80-year-old pulse is not as complex as a younger pulse since a single main peak is observed with a gradual decline. The similarities identified in the two previous pulse waveforms are present in these results. The single peak and the steady decline resulted in a slow increase in the duty values. The smooth diastolic phase of an older pulse was captured well with the pulse shaping, which was in stark contrast with the multiple-peak diastolic phase of the younger pulse. As a result, the MR pulse-shaping technique can also match elderly pulses. Therefore, the single-cam pulse-shaping method was successful in replicating a range of pulses from 20–80-year-olds.

The former discussion focused solely on the qualitative success of the pulse-shaping technique. A more quantifiable method of understanding the success of the technique is calculating the error between the experimental and in vivo pulse waveforms. This study used the root-mean-square error (RMSE) as a marker, the equation of which is found below.

(1) $$RMSE=\sqrt{\frac{{\left(exp-in\;vivo\right)}^{2}}{N}}$$

The RMSE was found by taking the difference between the experimental and in vivo data and then dividing it by the total number of data points, N (the values were squared and then square-rooted to ensure positive values were returned). The number of data points in the experimental data was reduced to match the number of data points from the in vivo data from 0 to 0.4 s. The RMSE values were found for all three ages, and they were all around 3%. Specifically, the errors were 3.7%, 3.3%, and 3.5% for the 20-, 50-, and 80-year-olds, respectively. This was a very low error overall, meaning the experimental pulses were representative of the in vivo pulses. The quantitative error values matched the qualitative findings, which found that the pulse-shaping method was successful for all age ranges.

The results demonstrate that the system can generate a wide range of radial pulses using a single cam disk, with the MR fluid effectively shaping the base pulse into various age-related patterns. To further improve the single-cam pulse shaping, this study next investigated a slope-based pulse-shaping method.

## 4. Slope-Based Pulse Shaping

**Figure 9:**
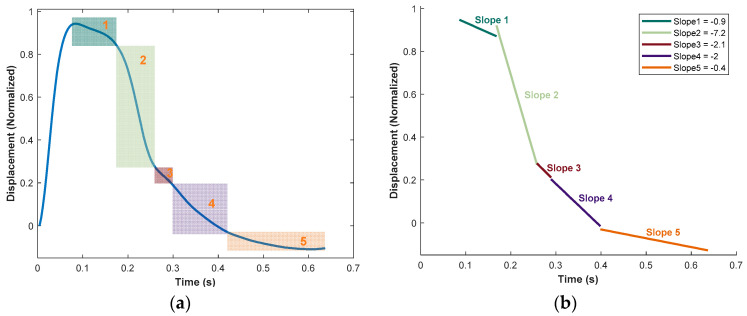
(**a**) Example of 5 slope zones analyzed in 80-year-old in vivo pulse; (**b**) 5 slopes that represent the 80-year-old in vivo pulse.

This section investigates the slope-based pulse-shaping method, aiming to develop a systematic control approach for the MR pulse simulator through a parametric study of the input values. The slope-based pulse technique involved calculating the slopes of the desired radial pulse during the descending phase and then determining the necessary inputs to replicate the pulse accurately. The initial steep ascension of the pulse was not calculated as the incline for normalized pulses is about the same, as can be seen in Figure 4b. To develop this method, the range of slopes required for age-related slopes was found. As noted before, the radial pulses of 20–80-year-olds can be used to represent the entire lifespan of a human. Therefore, the slopes of 20-, 50-, and 80-year-old radial pulses were determined by breaking each pulse into five regions. Each region was a relatively linear section which, when pieced together, was representative of the entire radial pulse. The slope analysis of the 80-year-old pulse can be found in Figure 9. An example of the five zones can be noted in Figure 9a. The resulting slopes of the five regions and the “pieced”-together 80-year-old pulse are shown in Figure 9b. The overall shape of the slope lines resembles the descending phase of an 80-year-old pulse. Once these slopes were determined, the range of slopes was found to be between 0 (or a horizontal line) and −7.2. This meant the experimental set-up must be able to replicate slopes anywhere from a horizontal line to a steep descent. Once the range of slopes was established, the next step was to investigate the multiple ways to obtain these slopes. Thus, a parametric study was conducted.

### Effects of Parametric Variations on Slope

**Figure 10:**
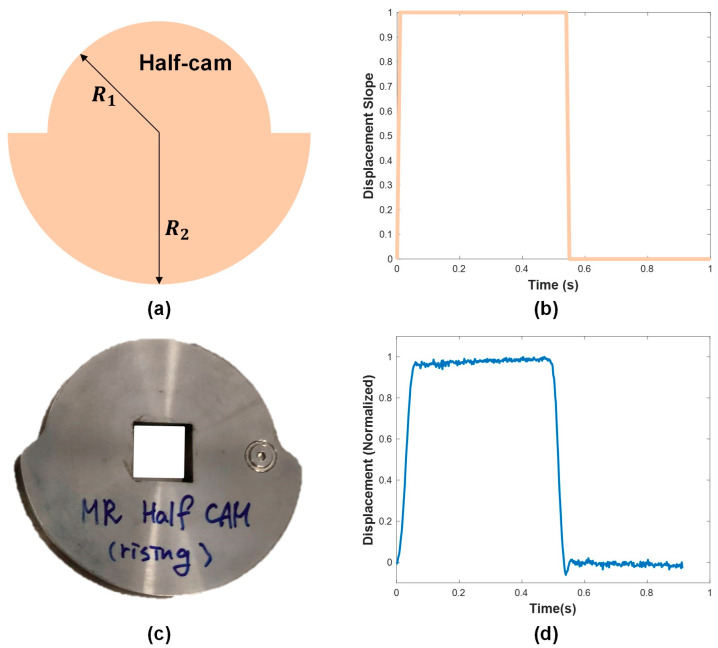
(**a**) Schematic design of half-cam, which maintains a constant radius for half of the disk; (**b**) normalized displacement graph of half-cam, illustrating constant displacement due to a constant radius.; (**c**) Fabricated actual half-cam using wire-cutting, (**d**) actual normalized displacement by half-cam.

The following section explains the parametric study that was performed to understand how the MR fluid shaped the base pulse. The goals of the parametric study included determining the capability of the system to generate a range of slopes within the required 0 to −7.2 range and the variables that affected the displacement slope. Note that the displacement slope refers to the slope of the pulse waveform generated by the movement of the plunger. Thus, the term “displacement slope” describes the gradient of the pulse waveform influenced by the plunger’s displacement. To better capture the effect of the MR fluid on the slopes, a specific half-cam was used in place of the base pulse cam. A schematic of the design of the half-cam is shown in Figure 10a, which maintains two distinct radii for half of the circumference. The theoretical normalized displacement of the half-cam is illustrated in Figure 10b, where the horizontal line displacement is due to the constant radius. The actual physical half-cam is shown in Figure 10c, with the actual normalized displacement in Figure 10d. The actual displacement matches well with the design, as there is a constant horizontal displacement over the cycle. The shape and displacement graph of the half-cam is in stark contrast with the base pulse input, which has various slopes throughout its phase. The advantage of using the half-cam for this parametric study was the ability to understand the direct impact the duty values had on the displacement slope. With the half-cam, any deviation in the slope was attributed to the duty values rather than the inherent shape of the cam disk. After choosing the half-cam, the main parameters of the duty values were determined. Duty values that would significantly alter the horizontal displacement slope were chosen. The electromagnet (which altered the state of the MR fluid) was controlled via the duty values, which, in part, changed the displacement. The initial testing showed a ramp input of duty values most affected the displacement. Using a ramp input of duty values, it was concluded there are three main ways in which the duty values can change: slope, magnitude, and time duration. These variables were investigated to determine which ones affected the displacement slope.

**Figure 11:**
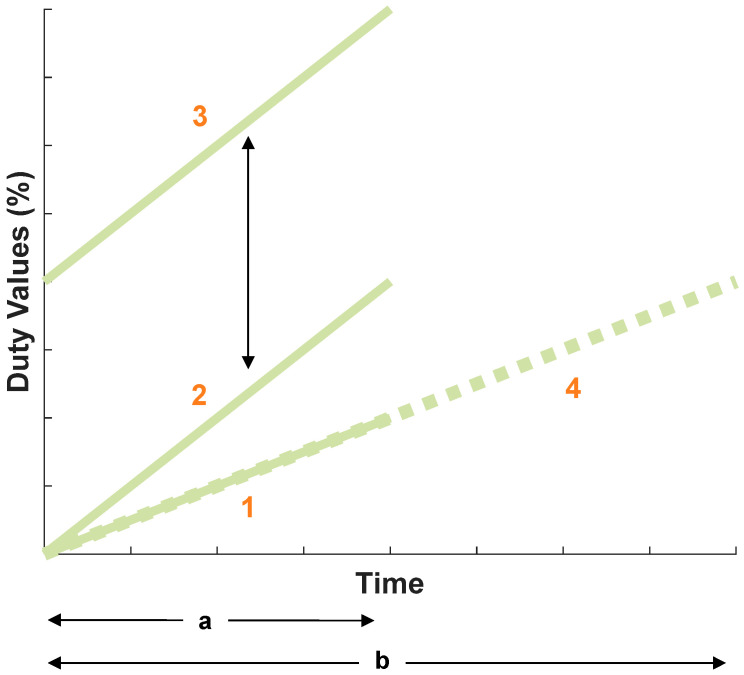
Graph showing three different variables tested during parametric study: duty slope, time duration, and magnitude.

Figure 11 illustrates the three variables related to the duty values. The slope variable is depicted by the difference between lines 1 and 2, with slope 2 being larger than slope 1. The duty slope, which represented the change in duty per millisecond, was calculated by dividing the percent increase in duty by the time period over which the duty values were applied (units: %/s). While the duration of the constant duty input remained the same, the initial duty value, which represented the electromagnetic force input waveform that determined the rising slope of the blood pressure waveform, was held constant. However, the final duty value changed from line 1 to line 2, resulting in an increased slope. The effect of changing the magnitude of the duty cycle is best illustrated between lines 2 and 3. While the slope between these lines remains consistent and the duration over which they act is the same, line 3 is shifted upward, increasing the magnitude of the duty values. Although the initial duty values between the lines differ, they maintain the same slope, which affects the magnitude of the duty values. The final variable examined was the time duration, observed between lines 1 and 4. Both lines exhibit a constant slope and start at the same duty value, but the duration for line 4 (b) is significantly longer than that for line 1 (a). Therefore, lines 1 and 4 highlight the difference in the time duration. These three variables are key methods for adjusting a ramp duty input. The parametric study isolated and varied these variables individually to predict the displacement slope across a range of duty values.

**Figure 12:**
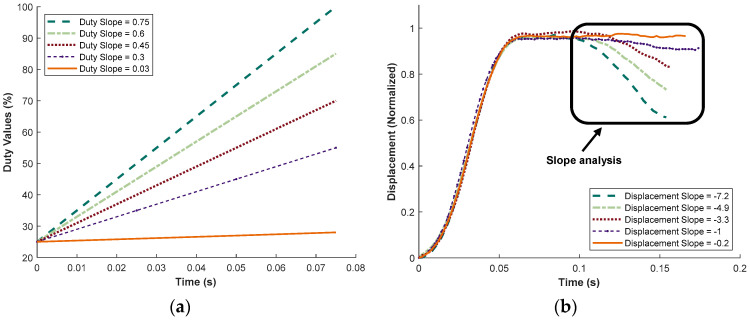
Results showing effect of changing duty slope on displacement slope. (**a**) Changing duty slope from 0.03%/s to 0.75%/s. (**b**) Resulting normalized displacement slope from changing duty slopes; displacement slopes are taken from within the boxed area.

The first of a series of three tests is described as follows. This initial study varied the duty slopes to observe their effect on the displacement slopes, with the results shown in Figure 12. The overall duration of the duty values remained constant at approximately 0.075 s (750 ms), and the initial duty value was kept at 25% for all slopes. This consistency was based on preliminary tests, which indicated that varying the duty slope had little effect on the displacement slope if the final duty value remained constant. Therefore, the initial duty slopes were kept constant to ensure more reliable results. The input duty slopes, ranging from 0.03%/ms to 0.75%/ms, are illustrated in Figure 12a. These variations in the duty slopes resulted in final duty values ranging from 28% to 100%. The color and line types in Figure 12a correspond to the results shown in Figure 12b, which depicts the normalized displacement of the half-cam for various duty slopes.

In Figure 12b, the initial ascent and horizontal plateau characteristics of the half-cam are visible. Duties were activated during the middle of the plateau, and the resulting descending slope represents the calculated slope value. To ensure that the descending displacement slope was attributable solely to the duty values, the duty values were delayed during the middle of the half-cam cycle for accurate measurements. The results indicate that the displacement slope ranged from 0 to −7.2, encompassing the range needed to represent various in vivo pulses throughout a human lifespan. Additionally, a direct relationship was observed between the duty slope and the displacement slope: as the duty slope increased, so did the displacement slope. A duty slope of 0.03%/ms had a minimal impact on the displacement slope (appearing as a horizontal line), while a duty slope of 0.75%/ms resulted in a displacement slope of −7.2. These findings demonstrate that the duty slopes significantly affected the normalized displacement slope. Although this test used an initial duty value of 25%, future tests explored the effect of varying the initial duty value, or the magnitude of the duty values.

**Figure 13:**
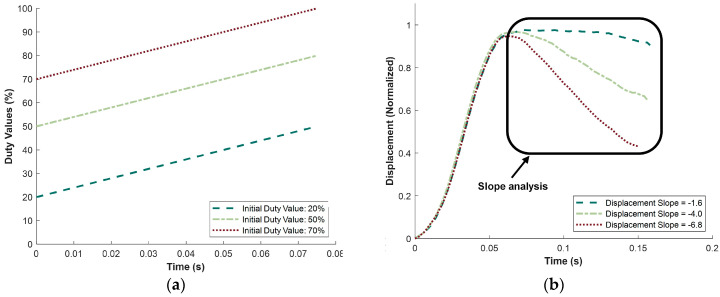
Results showing effect of changing duty magnitude on displacement slope. (**a**) Changing initial duty value from 20 to 70%. (**b**) Normalized displacement from changing duty magnitude; displacement slopes are taken from within the boxed area.

Once the range of displacement slopes was established, the effect of varying the initial duty value was investigated. The results of changing these initial duty values are shown in Figure 13. The initial duty values tested were 20%, 50%, and 70%, as illustrated in Figure 13a, with a constant duty slope of 0.4%/ms. The final displacement slopes are depicted in Figure 13b within the boxed area. The line types and colors match between the two graphs, showing that as the initial duty value increased, the displacement slope also increased. Specifically, there was a significant change in the displacement slope between the initial duty values of 20% and 70%. An initial duty value of 20% resulted in a minimal change in the displacement slope, while an initial duty value of 70% led to a displacement slope of nearly −7. This suggests there may be a threshold value where the MR fluid’s strength significantly alters the displacement slope. A threshold of around 50% was indicated by the significant displacement slope of −4 observed at this initial duty value. Furthermore, the displacement slope of −6.8 with a 0.4%/ms duty slope was very similar to the displacement slope of −7.2 with a 0.75%/ms duty slope. This implies that various combinations of slope and initial values can produce similar displacement slopes. The goal of this parametric study was to define these relationships and understand how the three variables—duty slope, initial magnitude, and time duration—can be adjusted simultaneously to achieve a desired displacement slope. The final test focused on altering the time duration of the duty values to determine its effect on the displacement slopes.

**Figure 14:**
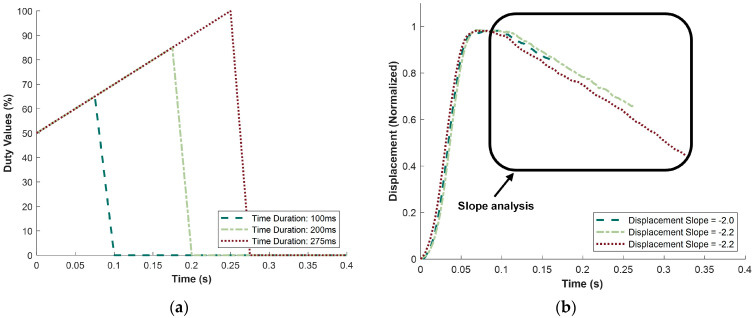
Results showing effect of changing time duration on displacement slope. (**a**) Changing total time duration from 0.100 to 0.275 s. (**b**) Normalized displacement from changing duty magnitude; displacement slopes are taken from within the boxed area.

The third and final test of the parametric study examined the effect of varying the duration for which the duty values were applied. The results of this testing are shown in Figure 14. The duration of the duty values varied between 100 ms, 200 ms, and 275 ms. The testing was constrained by the period of the half-cam, which allowed a maximum duration of 400 ms. As shown in Figure 14b, the displacement slope did not change with varying time durations; it remained close to −2, even when the duration of duty value activation nearly tripled. This indicates that only the previous two variables (duty slope and initial magnitude) significantly affected the displacement slope.

This parametric study established a direct relationship between the duty slopes and displacement slopes, with the displacement slopes ranging from 0 to −7.2. Additionally, the initial duty value significantly influenced the displacement slope, showing another direct relationship. However, the duration for which the duty values were applied did not affect the displacement slope. Therefore, the key variables to consider when determining the displacement slope are the duty slopes and initial duty values. The findings of this parametric study provide insights into how duty values influence displacement and help identify the variables that cause changes in pulse waveforms.

## 5. Conclusions

In this study, we aimed to design and test a cost-effective pulse simulator that can accurately replicate a wide range of age-dependent radial pulses. We integrated a magneto-rheological (MR) fluid device into a cam-based pulse simulator, which created a base pulse that was then shaped by the MR fluid. The comparison between the experimentally obtained pulse waveforms and the in vivo pulse waveforms showed an RMS error of less than 4% across all age groups, demonstrating the simulator’s capability to reproduce human radial pulse waveforms accurately. After confirming that the MR simulator could produce three representative in vivo pulses, we conducted a parametric study to demonstrate the feasibility of the slope-based pulse-shaping method for the MR pulse simulator. To extend the current slope-based control method for future work, we plan to develop a mathematical model that captures the control input and output slope relationship to predict the pulse displacement slope and produce a target pulse waveform. This model will then be experimentally verified with in vivo pulses. This will enable the simulator to continuously simulate arbitrary pulses in arteries, proving its potential for broader applications in healthcare.

## Data Availability

Data are contained within the article.
